# Early Introductions of *Candida auris* Detected by Wastewater Surveillance, Utah, USA, 2022–2023

**DOI:** 10.3201/eid3010.240173

**Published:** 2024-10

**Authors:** Jorge Chavez, Katherine Crank, Casey Barber, Daniel Gerrity, Thomas Iverson, Joshua Mongillo, Angela Weil, Linda Rider, Nathan Lacross, Kelly Oakeson, Alessandro Rossi

**Affiliations:** Utah Department of Health and Human Services, Salt Lake City, Utah, USA (J. Chavez, T. Iverson, J. Mongillo, A. Weil, L. Rider, N. Lacross, K. Oakeson, A. Rossi);; Southern Nevada Water Authority, Las Vegas, Nevada, USA (K. Crank, C. Barber, D. Gerrity)

**Keywords:** *Candida auris*, wastewater, surveillance, outbreak, fungi, Utah, United States

## Abstract

*Candida auris* is considered a nosocomial pathogen of high concern and is currently spreading across the United States. Infection control measures for *C. auris* focus mainly on healthcare facilities, yet transmission levels may already be significant in the community before outbreaks are detected in healthcare settings. Wastewater-based epidemiology (culture, quantitative PCR, and whole-genome sequencing) can potentially gauge pathogen transmission in the general population and lead to early detection of *C. auris* before it is detected in clinical cases. To learn more about the sensitivity and limitations of wastewater-based surveillance, we used wastewater-based methods to detect *C. auris* in a southern Utah jurisdiction with no known clinical cases before and after the documented transfer of colonized patients from bordering Nevada. Our study illustrates the potential of wastewater-based surveillance for being sufficiently sensitive to detect *C. auris* transmission during the early stages of introduction into a community.

*Candida auris* is an antifungal-resistant yeast that leads to high mortality rates among patients with underlying conditions and displays a formidable persistence in healthcare settings because of its biofilm-forming potential and resistance to some commonly used disinfectants (*1*–*3*). Circulating strains are classified into 5 genomic clades linked to their geographic area of origin: clade I (southern Asia), clade II (eastern Asia), clade III (Africa), clade IV (South America), and clade V (Iran) (*4*,*5*). Since *C. auris* introduction into the United States was documented in 2013 (*6*), prevalence has increased rapidly and the organism has become endemic to many jurisdictions (*7*). The strain exerted by the COVID-19 pandemic on infection control practices and public health resources in general is thought to have contributed to *C. auris* expansion (*8*).

Despite the widespread prevalence of *C. auris* in the United States, its effect on healthcare facilities should be limited, and jurisdictions that have not yet experienced sustained transmission should be protected. In addition to classic infection prevention strategies focused on admission screenings, point prevalence surveys (PPSs), and interfacility communication (*9*,*10*), wastewater-based surveillance could help control the spread of emerging pathogens by providing opportunities for early detection and management of outbreak responses (*11*–*13*).

We and others have previously shown the feasibility of community-scale wastewater surveillance for *C. auris* in high disease prevalence settings, specifically Nevada and Florida (*14*–*16*). We monitored the influent of the only wastewater treatment plant (WWTP) in St. George, Utah, before and after the transfer of a *C. auris*–positive patient into that community. On the basis of available epidemiologic information and modeling, we propose that wastewater surveillance could be a sufficiently sensitive strategy for early detection of *C. auris*, before it is detected in clinical surveillance efforts. In addition, we report improvements to the culture method that we originally used to recover *C. auris* isolates from wastewater and demonstrate the utility of organism isolation in providing high-quality genomic data for investigations. Our study was performed under Utah Department of Health and Human Services (DHHS) Institutional Review Board protocol no. 651 (“Community and facility level surveillance for multidrug resistant organisms using wastewater samples”).

## Materials and Methods

### Wastewater Sample Collection and Transport

**Figure 1:**
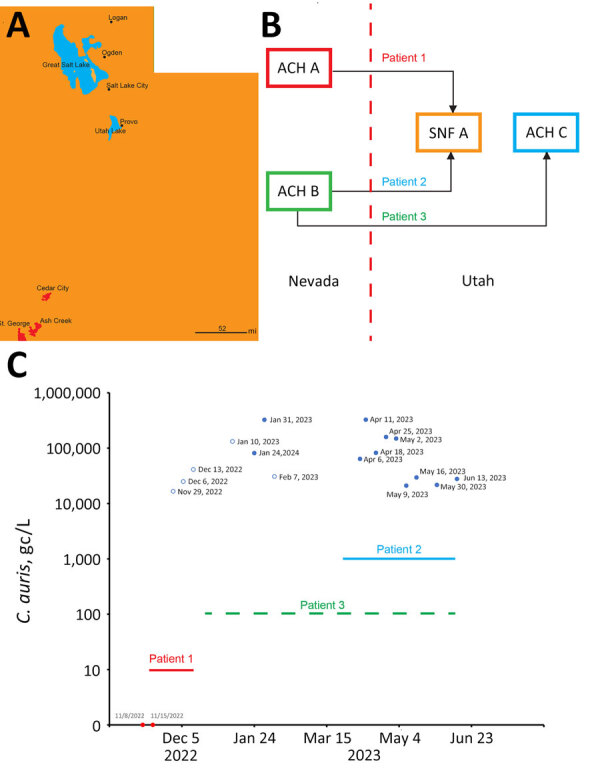
Transfer of *Candida auris* cases from Nevada to St. George, Utah, USA, and quantitative PCR monitoring of *C. auris* concentrations at the St. George wastewater treatment plant. A) Locations of the sewersheds of St. George, Ash Creek, and Cedar City (red) in Utah. The western border of Utah is adjacent to Nevada. Scale bar indicates 52 miles. B) Interstate transfers from Nevada to Utah of 3 patients with *C. auris* infection (red dash line represents the state border). C) Sampling dates and corresponding *C. auris* concentrations in wastewater treatment plant influent samples expressed as gc/L, over the time of the study. Nondetected samples are indicated as solid red dots, positive samples with concentrations less than the limit of quantification are indicated as empty blue dots, and positive samples with concentration at or equal to the limit of quantification are indicated as solid blue dots. The patient time frames are indicated by horizontal lines. The line for patient 3 is dashed to indicated that the person commuted continuously between Nevada and Utah. ACH, acute-care hospital; gc, gene copies; SNF, skilled nursing facility.

During November 2022–June 2023, we collected 24-hour composite influent wastewater samples from 3 WWTPs in southwestern Utah, near the border with Nevada: St. George (population ≈92,000), Ash Creek (≈25,000), and Cedar City (≈32,000) (Table 1; Figure 1, panel A). The St. George WWTP served as the primary experimental site, and the Ash Creek and Cedar City WWTPs served as presumptive negative control sites (Table 1). The average flow rate for the St. George WWTP during this study was 12.73 million gallons per day (mgd), which corresponds to 138 gallons per capita per day (gpcd). The per capita wastewater generation rate is similar to the national average of 132 gpcd (*17*). Each sample consisted of 250 mL of influent wastewater collected in polypropylene bottles and transported on ice (≈24 hours) to the Utah Public Health Laboratory (UPHL). Subsequently, 150-mL aliquots of each sample were shipped on ice with an overnight priority service (≈24 hours) to the Southern Nevada Water Authority laboratory.

**Table 1:** *Candida auris* qPCR and culture results from St. George, Ash Creek, and Cedar City, Utah, USA, 2022–2023*

Site	Sample date	qPCR Cq	No. qPCR amplifications (of 3 reps)	Quantifiable (LoQ = 33.03)	qPCR ESV, mL/reaction	qPCR concentration, log_10_ gc/L†	Culture results	Avg. WWTP flow, mgd
St. George	2022 Nov 8	‡	0	‡	1.13	<2.95‡§	Neg¶	11.67
	2022 Nov 15	‡	0	‡	1.13	<2.95‡§	Neg¶	11.55
	2022 Nov 29	33.42	3	No	3.75	4.22#	Neg¶	12.21
	2022 Dec 6	34.85	2	No	1.00	4.40#	Neg¶	11.89
	2022 Dec 13	34.78	2	No	0.75	4.61#	Neg¶	11.84
	2023 Jan 10	33.74	3	No	0.50	5.12#	Neg¶	12.47
	2023 Jan 24	30.96	3	Yes	1.50	4.91	NP	12.81
	2023 Jan 31	31.58	2	Yes	0.25	5.51	NP	12.75
	2023 Feb 7	33.53	3	No	0.75	4.49#	NP	12.67
	2023 Mar 28	NA	NA	NA	NA	NA	Pos**	14.08
	2023 Apr 4	NA	NA	NA	NA	NA	Pos**	14.06
	2023 Apr /6	31.70	3	Yes	0.38	4.81	NP	14.65
	2023 Apr 11	29.59	3	Yes	0.30	5.51	NP	13.98
	2023 Apr 18	29.22	3	Yes	1.50	4.92	Neg**	13.58
	2023 Apr 20	NA	NA	NA	NA	NA	Neg**	13.30
	2023 Apr 25	29.26	3	Yes	0.75	5.20	NP	13.17
	2023 May 2	30.38	3	Yes	0.38	5.17	NP	12.99
	2023 May 9	30.95	3	Yes	1.88	4.32	NP	12.53
	2023 May 16	31.21	3	Yes	1.13	4.47	NP	12.32
	2023 May 23	NA	NA	NA	NA	NA	Neg**	11.92
	2023 May 30	32.53	3	Yes	1.13	4.34	Neg**	11.87
	2023 Jun 13	32.18	3	Yes	1.13	4.44	Neg**	11.81
Cedar City	2023 Jan 24	‡	0	‡	1.50	<2.82‡§	Neg¶	3.26
	2023 Feb 7	‡	0	‡	0.37	<3.43‡§	NP	3.39
	2023 Jun 13	‡	0	‡	0.25	<3.60‡§	NP	2.99
Ash Creek	2023 Jun 13	‡	0	‡	0.75	<3.12‡§	NP	1.70

*Cq, quantification cycle; ESV, equivalent sample volume; gc, gene copies; LoD, limit of detection; LoQ, limit of quantification; mgd, million gallons/day; NA, samples not analyzed by qPCR; neg, negative; NP, not performed; pos, positive; qPCR, quantitative PCR; WWTP, wastewater treatment plant. †Concentration calculations were based on the average Cq of positive qPCR replicates. ‡Not detectable. §Left-censored concentrations are based on the theoretical LoD of 1 gene copy. ¶Culture performed via centrifugation method. #Reported concentrations are technically below the LoQ. **Culture performed via filtration method.

### Quantitative Real-Time PCR Monitoring and Performance Characteristics

To perform wastewater surveillance of *C. auris*, the Southern Nevada Water Authority used quantitative PCR (qPCR) as previously described (*15*). In the earlier study, the statistical limit of quantification was determined to be a quantification cycle (Cq) of 33.03 (*15*), which equated to an average of 7 gene copies (gc) across all study-specific standard curves, and the theoretical limit of detection was assumed to correspond to 1 gc. Across 18 samples in our study, the average equivalent sample volume (ESV) for each qPCR reaction was 1.07 ± 0.81 mL of influent wastewater, yielding an average limit of detection of 2.97 log_10_ gc/L. Limits of quantification ranged from 3.95 to 4.47 log_10_ gc/L based on variability in run-specific standard curves and an assumed average ESV of 1.07 mL (Appendix).

### *C. auris* Isolation by Culture of Filter-Based Concentration

We vacuum filtered <50 mL of influent wastewater through 0.45-μm cellulose nitrate analytical filters (ThermoFisher Scientific, https://www.thermofisher.com). We removed the filters from the vacuum unit by using forceps, placed the filters in a 50-mL conical tube, and submerged them in 12 mL of Salt Sabouraud Dulcitol Broth (*2*) (Thomas Scientific, https://www.thomassci.com) supplemented with 32 μg/mL fluconazole (*14*). The submerged filters were incubated at 42°C for up to 5 days with vigorous agitation at 250 rpm. We performed plating of the broth after incubation on chromogenic media and species identification of presumptive colonies as previously described (*14*).

### Whole-Genome Sequencing and Bioinformatics

We sequenced *C. auris* genomes by using a NextSeq platform (Illumina, https://www.illumina.com). We performed single-nucleotide polymorphism (SNP) distance analyses by using the MycoSNP pipeline (*18*), as previously described (*14*).

### Monte Carlo Modeling

We created a model to assess the theoretical sensitivity of the wastewater qPCR method for detecting *C. auris* from 1 shedding person in the sewershed. The model predicts the concentration of *C. auris* in wastewater (in gc/L) from 1 shedder (Table 2). We performed a Monte Carlo simulation by using 10,000 random samplings of the parameter distributions to characterize the distribution of possible *C. auris* concentrations with 1 *C. auris* shedder contributing urine and feces to the wastewater (Appendix).

**Table 2:** *Candida auris* shedding model parameters and distributions

Parameter	Unit	Reported value	Assumed distribution	Reference	Assumption
*C. auris* fecal shedding rate	CFU/μL	10^4^–10^5^	Uniform: min = 10^4^, max = 10^5^	(*19*)	Based on neutropenic mouse model
Daily wet stool production	g/day		Truncated: log-normal (base *e*): μ = 4.763, σ = 0.471, min = 0, max = 520	(*20*)	Based on healthy persons
Wet fecal density	g/mL	1.06	Point value: 1.06	(*21*)	NA
*C. auris* urine shedding rate	CFU/μL	10^2^–10^4^	Uniform: min = 10^2^, max = 10^4^	(*22*)	Based on the clinical definition of UTI for clean catch collection (e.g., >10^2^ CFU/μL)
Daily urine production	L/day		Gamma: shape = 5.315, scale = 0.25, offset = +0.5	(*23*)	Based on healthy persons
*C. auris* qPCR:culture (GC:CFU)	unitless	3–50	Uniform: min = 3, max = 50	(*24*)	Based on the analysis of several NCBI deposited *C. auris* genomes and a value reported for *C. albicans*
Average WWTP flow rate	mgd	12.73 ± 0.87	Normal: μ = 12.73, σ = 0.87	NA	NA

*GC, gene copies; mgd, million gallons/day; NA, no assumption made; NCBI, National Center for Biotechnology Information; qPCR, quantitative PCR; UTI, urinary tract infection; WWTP, wastewater treatment plant.

### Epidemiology Data and Infection Control Practices

*C. auris* reporting and submission of isolates or residual primary specimens is regulated by the Utah Communicable Disease Rule R386-702. The Utah DHHS uses EpiTrax as the centralized reportable disease database (*25*). Reports are filed electronically or via manual entry after notification to Utah DHHS by fax. Federal regulations recommend transfer notifications of patients colonized or infected with *C. auris*; however, compliance is seldom enforced, and effective interfacility communication relies on good infection prevention stewardship.

PPSs were conducted via composite axilla/groin swabbing (*26*) with nylon swabs, and samples were transported in liquid Amies (Eswab system; Copan, https://www.copanusa.com). We processed 200 μL of Amies media by using the on-board extraction PCR system BDMax (Becton, Dickinson and Company, https://www.bd.com) (*27*).

## Results

### Introduction of *C. auris* into St. George

In November 2022, the Utah DHHS was notified about the upcoming transfer of a patient with an active *C. auris* infection from Nevada to St. George, Utah (patient 1). Before patient 1 was transferred, no *C. auris* case or colonized person had been recorded in Utah. On November 10, 2022, patient 1 was transferred from an acute-care hospital (ACH A) in Nevada to a skilled nursing facility (SNF A) in St. George (Figure 1, panels B, C). Patient 1 was highly debilitated and while in St. George had a *C. auris*–positive urine culture (>10^5^ CFU/mL); the patient died after the transfer (December 2022) (Figure 1, panel C). No additional colonized persons were discovered at SNF A through a PPS evaluating 40 persons.

In March 2023, Utah DHHS was notified about the transfer of a second *C. auris*–colonized person from Nevada (patient 2). Patient 2 had been hospitalized in a different acute-care hospital in Nevada (ACH B) and was admitted to SNF A in St. George on March 23, 2023; the patient remained there for the duration of our wastewater surveillance study (Figure 1, panels B, C).

We discovered a third patient from Nevada (patient 3) retrospectively. Patient 3 previously resided at ACH B in Nevada (similar to patient 2) but was not found to be colonized with *C. auris* before being transferred to St. George in December 2022 (Figure 1, panels B, C). Because of the risk factor associated with patient 3 being transferred from a healthcare facility with an ongoing outbreak (i.e., ACH B in Nevada), the acute-care hospital in St. George (ACH C) ordered an admission screening, which led to confirmation of *C. auris* colonization on December 28, 2022 (Figure 1, panels B, C). The colonization status of patient 3 was communicated via fax to the Utah DHHS according to Utah communicable diseases rules; however, the alert was overlooked because of human error. After 2 days at ACH C, patient 3 was discharged and continued to receive dialysis in an outpatient setting in St. George until June 2023, albeit by commuting between his residence in Nevada and St. George (Figure 1, panel C).

Collectively, the 3 patient transfers potentially resulted in nearly continuous shedding of *C. auris* into St. George wastewater during November 2022–June 2023, which coincided with the duration of our wastewater surveillance study. Moreover, patients 2 and 3 simultaneously resided or spent a substantial amount of time in St. George during March–June 2023 (Figure 1, panel C), potentially increasing *C. auris* loading in local wastewater.

### Detection of *C. auris* in St. George Wastewater

After being notified of the pending transfer of patient 1 to St. George, we identified a unique opportunity to assess the sensitivity of wastewater surveillance for the early detection of *C. auris*. On November 8, 2022 (2 days before the transfer), we initiated qPCR-based wastewater surveillance at the St. George WWTP (Figure 1, panel C), which continued at irregular intervals until June 13, 2023 (Table 1; Figure 1, panel C). *C. auris* was not detected (i.e., below the limit of detection) in the first 2 samples collected for the study (November 8 and 15), which straddled the transfer date of patient 1 (Table 1; Figure 1, panel C). However, *C. auris* was detected, albeit below the limit of quantification, in a sample collected on November 29 and was detected in every sample thereafter (Table 1; Figure 1, panel C). Before March 2023, only 2 of 7 samples were above the limit of quantification, but starting in April 2023, when patients 2 and 3 were potentially contributing to the St. George WWTP, all samples were above the limit of quantification (Table 1; Figure 1, panel C).

To assess potential transmission in areas near St. George, we also analyzed 4 influent wastewater samples from WWTPs in Ash Creek and Cedar City (Figure 1, panel A). *C. auris* was not detected in those presumptive negative control samples (Table 1).

### Modeled Sensitivity of qPCR-based, Community-Scale Wastewater Surveillance

Given the available epidemiologic data and the fact that *C. auris* was not detected in the sample collected before the initial transfer of patient 1, the subsequent *C. auris*–positive wastewater could represent detection of its initial introduction in St. George. If true, that finding indicates that qPCR-based wastewater surveillance may be sufficiently sensitive to detect a single *C. auris* shedder in a sewershed serving ≈100,000 inhabitants.

To gauge the plausibility of that statement, we used a Monte Carlo simulation model previously used for SARS-CoV-2 but adapted to *C. auris* (*28*). The model considers the following parameters: *C. auris* concentration ranges for urine and feces (based on a neutropenic mouse model and quantitative analyses of urine clinical cultures [*19*,*22*]); urine and feces production rates in healthy humans (*20*,*21*,*23*); variable gc numbers of the qPCR target (internal transcribed spacer 2) across *C. auris* strains (based on a nucleotide BLAST analysis in which the sequence of the qPCR probe was searched in various *C. auris* genomes, as well as the number of rRNA genes reported in *C. albicans* as an upper hypothetical value [*24*,*29*,*30*]); and average daily wastewater flow rate (Table 2).

The primary site of *C. auris* colonization is skin (*31*), and routine hygiene practices (e.g., handwashing, showering, laundering) should also represent a major route for release of organisms into the sewer system. However, we did not incorporate that shedding mode in our model because it would entail assumptions with considerable uncertainty (e.g., affected water volumes, affected skin surface area, skin mobilization rate, frequency of handwashing/showering/laundering).

**Figure 2:**
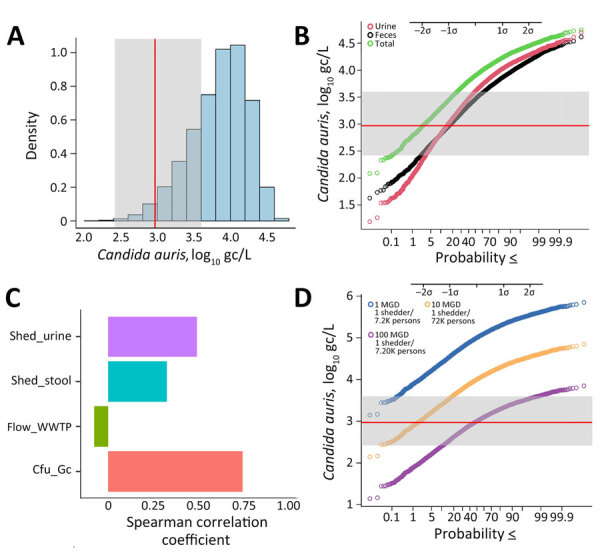
Monte Carlo simulation model forecasting *Candida auris* concentrations as a function of variable shedding levels in urine and feces, organism gene copy numbers, and WWTP flow rate (Table 2) as part of a study of *Candida auris* detection by wastewater surveillance, Utah, USA. A) Density plot of computed *C. auris* concentrations in wastewater resulting from 1 person shedding the organism in urine and feces. B) Probability plot showing the probability of detection at different organism concentrations. C) Sensitivity analysis, showing the correlation between individual parameters and their effect on predicted *C. auris* concentrations (i.e., the strength of the effect of each parameter on the model). D) Probability plot showing the effect of the magnitude of hypothetical flow rate (or sewershed size) on the likelihood of detecting the organism by quantitative PCR; infection prevalence metrics assume a St. George wastewater generation rate of 138 gpcd. In panels A, B, and D, the average limit of detection of the quantitative PCR is shown as a red line, and the minimum and maximum limits of detection observed in the study resulting from variation in sample-specific ESV are delineated by the gray area. Probabilities are less than or equal to the values indicated. ESV, equivalent sample volume; gc, gene copies; gcpd, gallons per capita per day; mgd, million gallons per day. WWTP, wastewater treatment plant.

Our model indicates that 97% of the predicted *C. auris* wastewater concentrations resulting from 1 person shedding the pathogen in urine and feces were above the average limit of detection for our study (2.97 log_10_ gc/L; assumes a limit of 1 gc and the average of all sample-specific ESVs for our study) (Figure 2, panels A, B). The median predicted concentration from the Monte Carlo simulation was 3.90 log_10_ gc/L, a value greater than the upper limit of observed limits of detection (i.e., 3.60 log_10_ gc/L; assumes a limit of 1 gc and the average of all sample-specific ESVs for our study) (Figure 2, panel A). The upper-bound probability of detection increases to 99.9% when the lowest limit of detection is considered (Figure 2, panel B). If shedding is modeled through either urine or feces alone, the probability of detection decreases to ≈85% when the average limit of detection is considered (Figure 2, panel B).

To determine the effect of each stochastic parameter on the final predicted wastewater concentrations, we used the Spearman correlation coefficient to perform a sensitivity analysis on the Monte Carlo model (*28*) (Figure 2, panel C). The parameter with the strongest correlation was the number of genome copies per CFU, followed by the rate of *C. auris* shedding in urine (and to a slightly lesser extent, feces). The flow rate had a moderate negative correlation, indicating that an increased flow rate results in a decreased probability of detection in wastewater from 1 shedder. To better illustrate this effect, we generated probability plots for 3 hypothetical WWTP flow rates: 1, 10, and 100 mgd. Assuming the St. George per capita wastewater generation rate of 138 gpcd, those flow rates represent *C. auris* infection prevalences of 1 in 7,200 (flow rate 1 mgd), 1 in 72,000 (flow rate 10 mgd), and 1 in 720,000 (flow rate 100 mgd) persons. At the average limit of detection, the detection probability relative to a single shedder was almost 100% at a flow rate of 1 mgd, ≈97.5% at 10 mgd, and 50% at 100 mgd (Figure 2, panel D). Altogether, our modeled and observed results support the hypothesis that use of qPCR-based wastewater surveillance for *C. auris* can achieve sensitivity on the order of 1 in 100,000.

### Isolation of *C. auris* from the St. George Sewershed and Genetic Relatedness to Clinical Isolates

**Figure 3:**
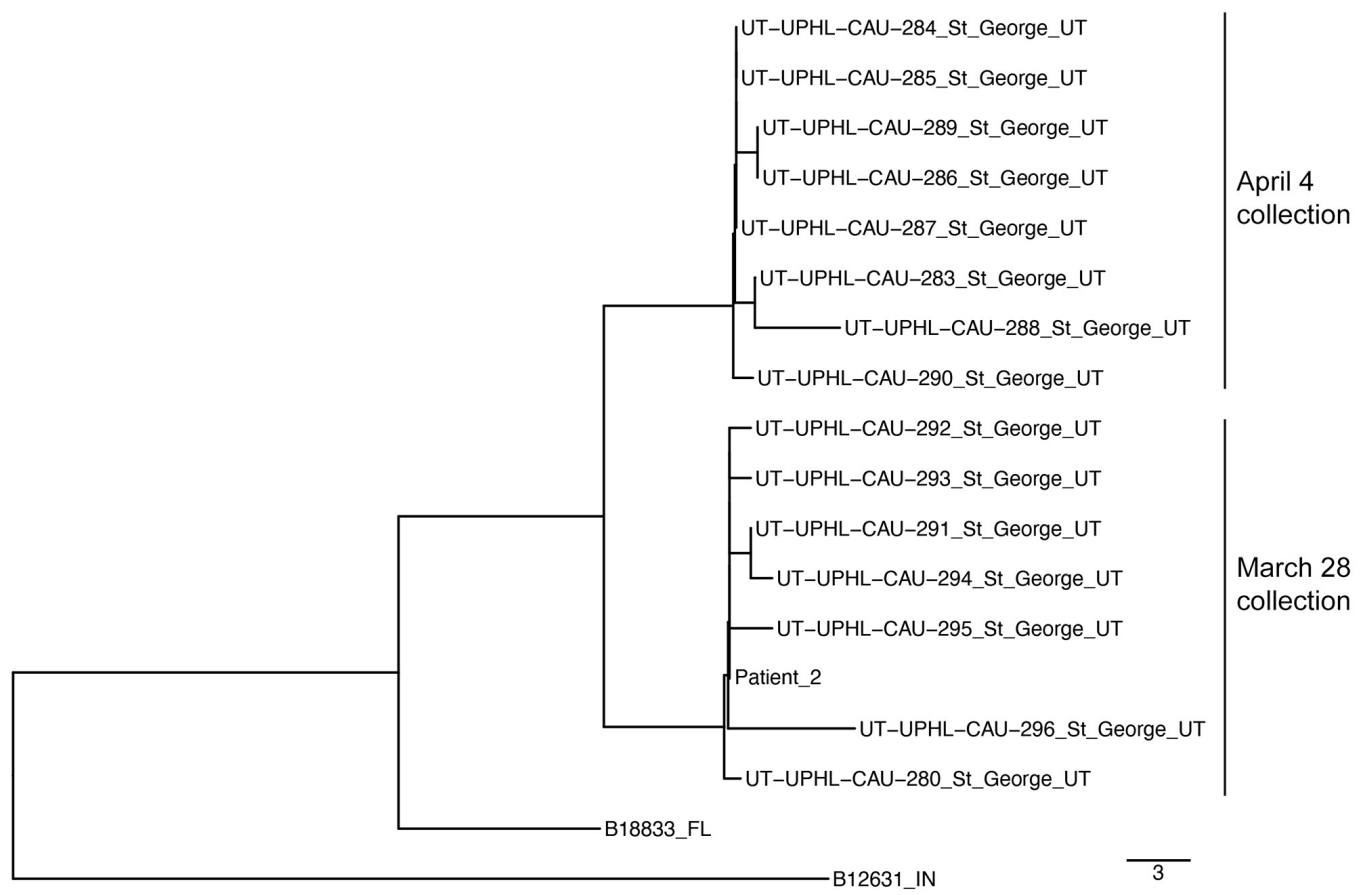
Neighbor-joining phylogenetic tree including clade III *Candida auris* isolates recovered from the St. George, Utah, USA, wastewater treatment plant on 2 collection dates (March 28 and April 4, 2023) and from a second patient. National Center for Biotechnology Information Sequence Read Archive accessions for all isolates are listed in the Appendix. Scale bar indicates single nucleotide polymorphism.

We complemented qPCR-based wastewater surveillance with culturing and subsequent whole-gene sequencing of recovered isolates. Initial attempts with the centrifugation-based method previously used for southern Nevada wastewater (*14*) were unsuccessful (Table 1). As such, we explored a filtration-based alternative that was observed to be superior to our original method across 2 split samples from Nevada (Appendix Figure 1). Using that improved method, we were able to recover 15 *C. auris* isolates from 2 samples consecutively collected on March 28, 2023, and April 4, 2023 (Table 1). All wastewater isolates belonged to clade III and segregated topologically into 2 individual subgroups distinctly separated by collection date (Figure 3). Isolates within the subgroup linked to the March 28 wastewater sample were highly related to the clinical isolate available for patient 2 (0–6 SNPs), who was transferred to St. George on March 23. The isolates within the subgroup linked to the April 4 sample displayed ≈12 SNP differences from the patient 2 isolate (Figure 3). Unfortunately, no clinical whole-genome sequencing (WGS) data were available for patient 3 because the patient’s colonization status was not determined in Nevada and the positive clinical sample collected in Utah was not submitted to UPHL. Patient 1 was infected with a clade I strain, but we were unable to culture *C. auris* in any wastewater samples collected during the time of the patient’s stay at SNF A or before March 28, 2023 (Table 1). As such, we were unable to study the contribution of clade I isolates to the overall *C. auris* signal in the St. George sewershed.

## Discussion

The recent history of *C. auris* in the United States highlighted the challenges in controlling the pathogen after it becomes established in an area (*7*). Early detection strategies coupled with aggressive infection control measures could reduce its effect on healthcare facilities and the general population. In that respect, wastewater-based surveillance is a promising tool for detecting pathogens that are circulating in the population at very low prevalence and have not been overtly manifested at the clinical level (*12*).

The application of wastewater-based surveillance to the *C. auris* problem is in its infancy (*14*–*16*), so fundamental parameters that will guide its use have not yet been studied in detail. For example, levels of *C. auris* shedding in human excreta and body site densities during colonization have not yet been adequately characterized. *C. auris* effectively colonizes skin and nares (*26*,*31*) but is not typically recovered from the buccal mucosa (*31*,*32*). A regular nylon swab can usually recover 10^2^–10^10^ CFU of *C. auris* from various skin sites (e.g., palms/fingertips, toe web, perianal skin, axilla, inguinal crease, neck) and nares (*31*). As such, it is conceivable that the organism could be released in great numbers into the sewershed via skin shedding during routine hygiene practices or even when laundering items that have been in contact with a colonized person. Because skin is the primary *C. auris* colonization site, clinical studies aimed at determining the actual bioburden released through hygiene practices (*33*) will be essential for assessing quantitative measurements in wastewater. *C. auris* is also commonly recovered from urine (*4*,*26*) of patients with candiduria (*34*), as well as from asymptomatic persons (*35*). *C. auris* is less frequently recovered from fecal samples but has been recovered via rectal swabbing (*26*,*32*,*36*). Of note, a correlation between gut colonization and urinary tract infections has been observed in cohorts of patients affected by *C. auris* (*36*).

As has been accomplished for wastewater-based surveillance of SARS-CoV-2, additional modeling and parameterization are needed to fully characterize relationships between incidence/prevalence and expected wastewater concentrations (*28*,*37*). Early attempts to establish those correlations for *C. auris* have been extremely challenging (*16*). Nevertheless, the qPCR data and the excreta-only model used in our study fit very well with the clinical course of patient 1 (the putative introduction event in St. George) and indicate that detecting 1 *C. auris* shedder to a community-scale wastewater system of moderate size is plausible (Figure 2). Although our study focused on early detection of pathogen introduction, future studies should consider monitoring wastewater *C. auris* loads after the population presumably returns to a zero-infection status.

Recovery of *C. auris* in culture has been instrumental in obtaining isolates for molecular epidemiology analyses by WGS (*14*). The incorporation of WGS into wastewater-based surveillance systems for *C. auris* should be universally adopted to understand the origin of introduction events as well as the evolving diversity of contributions to sewersheds. Motivated by the initial inability to recover *C. auris* isolates in St. George (Table 1), we worked at improving our original culture method by changing the sample concentration step from centrifugation to membrane filtration (Appendix Figure 1), an approach also recently used by Babler et al. (*16*). Yet, culture from wastewater samples remains a highly variable endeavor, possibly because of variable competition from other species of fungi or fluctuations in environmental factors within the sewer environment affecting the growth of *C. auris*, such as dissolved oxygen concentration (*38*,*39*). In addition, our broth enrichment approach remains unsuitable for isolating fluconazole-susceptible isolates (*14*).

WGS analysis indicated a close relationship between *C. auris* wastewater isolates and 1 isolate from patient 2 (Figure 3). When those wastewater samples were collected, both patients 2 and 3 were potentially contributing *C. auris* to the St. George WWTP (Figure 1, panel C). With the data available, we cannot discriminate whether the genetic diversity of the wastewater isolates collected on 2 separate dates encompasses shedding from patient 3 or other unidentified colonized persons. Moreover, mixed colonization consisting of clones separated by SNP distances greater than those displayed in Figure 3 is not unusual (*40*,*41*) and represents another layer of complexity in the interpretation of molecular epidemiology analyses for *C. auris* (*42*). However, incorporating WGS analyses into *C. auris* wastewater surveillance would still be invaluable for detecting contributions from strains belonging to different clades or displaying very large SNP distances. In addition, if *C. auris* strains can persist in sewer pipes as biofilm (a phenomenon not yet investigated for this organism) (*38*), WGS could potentially distinguish persistent signals from a new shedding event.

In conclusion, we used a holistic approach to *C. auris* wastewater-based surveillance that entailed using qPCR as the main testing method, as well as culture and WGS to better characterize the source of the molecular signals. After being proven effective, metagenomic approaches could potentially bypass the need for culture (*43*). We believe that our case study illustrates the potential of wastewater-based surveillance to be a sufficiently sensitive method for discovering *C. auris* transmission at early stages of introduction into a community.

Appendix Additional information for study of early introductions of *Candida auris* detected by wastewater surveillance, Utah, USA, 2022–2023.
