# Supplementary material for: Early Introductions of Candida auris Detected by Wastewater Surveillance, Utah, USA, 2022–2023
Source: Emerg Infect Dis. 2024 Oct;30(10):2108–17. doi: 10.3201/eid3010.240173 (PMC11431928; doi:10.3201/eid3010.240173)
Supplement: Appendix — Additional information for study of early introductions of Candida auris detected by wastewater surveillance, Utah, USA, 2022–2023. [file 24-0173-Techapp-s1.pdf]

Article DOI: https://doi.org/10.3201/eid3010.240173

EID cannot ensure accessibility for supplementary materials supplied by authors. Readers who have difficulty accessing supplementary content should contact the authors for assistance.

Early Introductions of *Candida auris* Detected by Wastewater Surveillance, Utah, USA, 2022–2023

Appendix

qPCR information

qPCR analysis of *Candida auris* in wastewater followed a previously published method (*1*). Briefly, 150-mL wastewater samples were centrifuged at 3200×*g* for 30 min, the supernatant was discarded, and nucleic acid extraction was performed on a portion of the pelleted solids using the DNeasy Power Soil Pro Kit (QIAGEN, https://www.qiagen.com/us). Using a sterile spatula, 0.1–1.0 g of pellet was transferred to the DNeasy Power Soil bead tubes, which contain mixed zirconium beads. Cell lysis was achieved using the kit's lysis buffer in conjunction with 10 min of vortexing at maximum speed, according to manufacturer's instructions. qPCR reactions were run in triplicate, with each reaction containing 2.5 µL of extracted DNA, 1× iTaq Universal Probes Supermix (BIO-RAD Laboratories, Inc., Hercules, CA), 0.50 µM of each primer (Forward: 5′-CAG ACG TGA ATC ATC GAA TCT - 3′, Reverse: 5′-TTT CGT GCA AGC TGT AAT TT-3′), 0.1 µM of probe (Probe: 5′-FAM-AAT CTT CGC GGT GGC GTT GCA TTC A−BHQ_1–3′), and sufficient nanopure water to bring the total reaction volume to 10 µL (*2*). The thermocycler process commenced with an initial denaturation stage at 95°C for 20 seconds. This was followed by 45 cycles of denaturation at 95°C for 3 seconds and annealing at 60°C for 30 seconds each. Standard curves were generated using gBlocks gene fragments (Integrated DNA technologies, http://idtdna.com). The equivalent sample volume (ESV), or the volume of wastewater assayed, was calculated from the various concentration, extraction, and qPCR steps as shown in Equation 1.

(Eq. 1)

$$ESV\left(\frac{mL}{rxn}\right) = \frac{template\ volume(\mu L/rxn)}{extraction\ elution\ volume(\mu L)} \times \frac{pellet\ used(mL)}{pellet\ total(mL)} \times starting\ volume\ (mL)$$

Monte Carlo modeling equation and assumptions

The predicted concentration of *C. auris* in wastewater (in gc/L) from a single shedder was defined as follows:

$$C_{Caur}=\frac{\left(Caur_{feces}\times\frac{Mass_{feces}}{\rho_{feces}}+Caur_{urine}\times V_{urine}\right)}{Q_{WW}}\times(GC:CFU)\ \text{(Eq. 2)}$$

where,

C_{Caur} = Modeled concentration of *C. auris* in wastewater from a single shedding individual (gc/L)

$Caur_{feces}$ = *C. auris* fecal shedding rate (CFU/L)

$Mass_{feces}$ = Mass of feces produced per day by the shedding individual (g/day)

ρ_{feces} = density of feces produced by the shedding individual (g/L)

$Caur_{urine}$ = *C. auris* urine shedding rate (CFU/L)

V_{urine} = Volume of urine produced per day by the shedding individual (L/day)

Q_{ww} = Wastewater treatment plant flow rate (L/day)

GC:CFU = *C. auris* ITS2 gene copies (GC) per colony forming unit (CFU) (unitless)

Observed average daily flow rates for the St. George WWTP were fit to a normal distribution in R using the 'fitdistrplus' package (*3*).

References

1. Barber C, Crank K, Papp K, Innes GK, Schmitz BW, Chavez J, et al. Community-scale wastewater surveillance of *Candida auris* during an ongoing outbreak in Southern Nevada. Environ Sci Technol. 2023;57:1755–63. PubMed https://doi.org/10.1021/acs.est.2c07763

2. Leach L, Zhu Y, Chaturvedi S. Development and validation of a real-time PCR assay for rapid detection of *Candida auris* from surveillance samples. J Clin Microbiol. 2018;56:e01223–17. PubMed https://doi.org/10.1128/JCM.01223-17

3. Delignette-Muller ML, Dutang C. fitdistrplus: An R Package for Fitting Distributions. Journal of Statistical Software. 2015;64:1–34. https://doi.org/10.18637/jss.v064.i04

Appendix Table. SRA accessions for the isolates included in the phylogenetic tree shown in Figure 3 in the main article.

Isolate	SRA accession
Patient_2	SRR27596512
UT-UPHL-CAU-288	SRR25757299
UT-UPHL-CAU-287	SRR25757298
UT-UPHL-CAU-286	SRR25757293
UT-UPHL-CAU-285	SRR25757292
UT-UPHL-CAU-284	SRR25757291
UT-UPHL-CAU-283	SRR25757290
UT-UPHL-CAU-296	SRR25757289
UT-UPHL-CAU-295	SRR25757288
UT-UPHL-CAU-294	SRR25757287
UT-UPHL-CAU-293	SRR25757286
UT-UPHL-CAU-292	SRR25757297
UT-UPHL-CAU-291	SRR25757296
UT-UPHL-CAU-290	SRR25757295
UT-UPHL-CAU-289	SRR25757294
UT-UPHL-CAU-280	SRR24486935
B12631_IN	SRR7909359
B18833_FL	SRR12526241

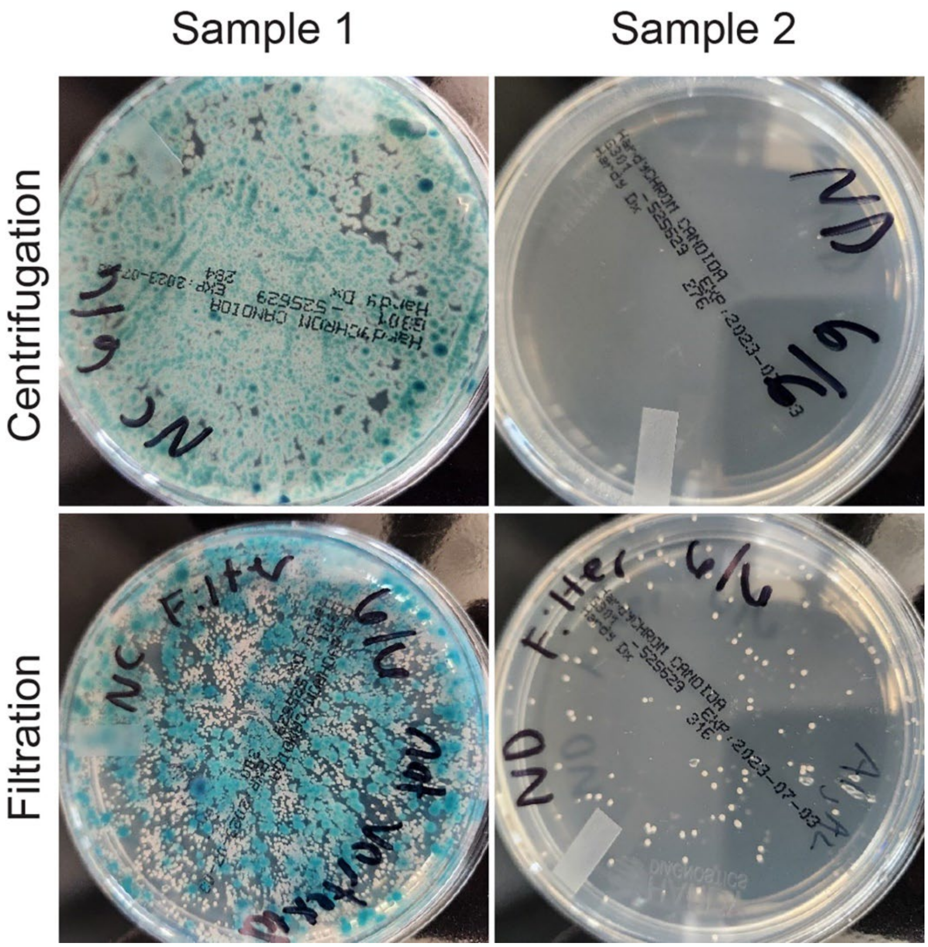

Appendix Figure. Individual wastewater samples from two different sites in Nevada (Sample 1 and Sample 2) were split to compare the effectiveness of *C. auris* recovery by culture using either centrifugation or membrane filtration as a concentration step. The centrifugation method (Rossi et al., 2023) entailed spinning the wastewater sample at 5,000 x g for 10 minutes and resuspending the resulting pellet in 0.9% 1 mL of saline. 100 µL of the resuspended pellet was used to inoculate 2 mL of SSDBF (32 µg/mL fluconazole). As described in material and methods, the filtration method entails running a wastewater sample until it has flown through the membrane. The membrane is then removed and transferred in a 50 mL conical tube where it is entirely submerged with 12 mL of SSDBF. Broth enrichment conditions (up to 7 days at 42°C with 250 rpm shaking), plating volumes (100 µL), and selective/differential media [HardyCHROM Candida medium (Hardy Diagnostics, https://hardydiagnostics.com) or BBL CHROMagar Candida medium (Becton, Dickinson and Company, https://www.bd.com)] were identical in both methods. For sample 1 the starting volume was 25 mL and 26 mL for sample 2. Sample 1 processed via centrifugation yielded only colonies of a competing fungal species (blue). When Sample 1 was processed via filtration numerous isolated *C. auris* colonies (lilac) grew together with the contaminant. Sample 2 processed via centrifugation did not yield any growth. When Sample 2 was processed via filtration pure *C. auris* growth was obtained.
